# Improved Value of Individual Prenatal Care for the Interdisciplinary Team

**DOI:** 10.1155/2018/3515302

**Published:** 2018-09-17

**Authors:** Ella Damiano, Regan Theiler

**Affiliations:** Department of Obstetrics and Gynecology, Dartmouth-Hitchcock Medical Center, Lebanon, NH, USA

## Abstract

**Objective:**

Innovative models of prenatal care are needed to improve pregnancy outcomes and lower the cost of care. We sought to increase the value of traditional prenatal care by using a new model (PodCare) featuring a standardized visit schedule and coordination of care within small interdisciplinary teams in an academic setting.

**Methods:**

Prenatal providers and clinic staff were divided into four “Pods”. Testing and counseling topics were assigned to visits based on gestational age. Interdisciplinary weekly Pod meetings provided coordination of care. A retrospective chart review was performed. The primary endpoints were the number of prenatal care visits and number of providers seen.

**Results:**

After PodCare implementation, more patients choose care with the low-risk physician team (42% compared to 26%). Study subjects included 85 women in 2013 and 165 women in 2014. The median number of visits decreased from 13 to 10 (p < 0.00004) and the median number of providers seen decreased from 7 to 5 (p < 0.0000008).

**Conclusion:**

PodCare increased the value of individual prenatal care by decreasing the number of visits, increasing continuity, and providing care coordination. The model provides a robust experience in interdisciplinary care. The PodCare model may be successful at other academic institutions.

## 1. Introduction

Prenatal care seeks to mitigate risks and promote positive maternal and neonatal outcomes [1]. There is limited evidence for the best model of care. The American College of Obstetricians and Gynecologists (ACOG) recommends prenatal visits every four weeks until 28 weeks; then every two weeks until 36 weeks and weekly until delivery [2]. This schedule is not data driven and increased frequency of prenatal visits does not correlate with improved outcomes [3].

National objectives and quality measures, such as Healthy People 2020 and the Healthcare Effectiveness Data and Information Set (HEDIS), feature similar goals for quality prenatal care. These goals include improved timeliness of care and adequate attendance to visits and postpartum care [4, 5].

In this retrospective cohort study, we tested the hypothesis that a new model of prenatal care, PodCare, would increase value by decreasing the number of visits while increasing continuity with providers and maintaining current high quality care.

## 2. Methods

This retrospective cohort study analyzed data obtained at Dartmouth-Hitchcock Medical Center in 2013 and 2014. This study was IRB approved (#28728) at Dartmouth College. The writing of this article followed the Standards for Quality Improvement Reporting Excellence (SQUIRE) 2.0 [6].

At Dartmouth-Hitchcock Medical Center, a rural tertiary care academic medical center in Lebanon, NH, women received prenatal care from a number of providers including academic generalists, maternal-fetal medicine (MFM) specialists, and midwives. Patient enrollment in physician or midwife care is a voluntary decision; care is provided by the MFM team when indicated by the patient's condition. The physician team is composed of attending physicians, associate providers including advanced practice nurses and midwives, and obstetrics and gynecology resident physicians.

Prior to 2013 attempts to form smaller teams of providers were unsuccessful due to challenges with scheduling and lack of cohesion within teams. Therefore, providers acted as one large team and patients were scheduled with any of approximately 25 providers for a visit. This model did not encourage coordination of care or continuity with providers. The last provider seen would determine the timing of the next appointment, roughly following ACOG guidelines. Under this traditional care model, there was lack of care coordination and continuity due to visits with multiple providers.

In 2014, a new model for small team-based physician care, “PodCare,” was designed. Four teams, or “Pods,” were created whose members included physicians, associate providers, a nurse, and a secretary. Resident physicians were assigned a Pod at the beginning of residency and continued with that Pod throughout the four years. Pods were led by 1-2 attending physicians, and each Pod had one associate provider, such as an advanced practice nurse or a nurse midwife. A designated nurse and secretary were also assigned to the Pod team. Patients were given a business care for the Pod, which included all the names of all providers, including the nurse, and the secretary's phone number. Patients were also introduced to their Pod secretary at checkout, where signage indicated each Pod's team members. There were no other major changes to prenatal care or hospital obstetric practice at this time.

Key changes of the PodCare model included emphasis on team continuity for all appointments, adherence to a structured schedule of appointments, and weekly Pod meetings to monitor patient care plans. Patients were informed of the PodCare model at the new obstetrical appointment and had the option to choose physician PodCare, Centering Pregnancy®, or midwife individual care.

All prenatal visits were individual office-based prenatal care. Ten prenatal visits are expected if a pregnancy continues to 40-week gestation with an additional visit for those requiring late term induction of labor. This schedule represents a decrease in the number of visits from ACOG's Guidelines for Perinatal Care, [2] which would recommend 14 visits to reach the due date. Residents' schedules throughout the year, with the exception of night float, include a weekly continuity clinic to care for Pod patients. Each Pod had the capacity to care for approximately 100 women per year.

In addition to revising the patient's schedule of care, this model required changes to providers' schedules. Weekly resident physician didactic schedules were extended to include one hour of Pod meeting. Clinic appointments were not scheduled during that hour to allow for inclusion of all associate providers, nurses, and secretaries. At the weekly Pod meetings, providers select patients for discussion to ensure completeness of care and appropriate delivery planning.

At Pod meetings, less experienced clinicians, such as junior residents, can request help from experienced clinicians, such as senior residents, while still being supervised by the attending physician. Between meetings, providers can generate interim care plans by messaging through the electronic medical record with the Pod attending. Patients interact with the same providers, secretary, and nurse throughout care.

For this study, data were abstracted retrospectively from the electronic medical record (Epic) for all patients initiating prenatal care in the one year before and after PodCare. A washout period of six months on either side of the intervention was applied. This resulted in a study period of January 1-June 30, 2013, and July 1-December 31, 2014. Study subjects were eligible if their first obstetrical appointment was during the study period.

Inclusion criteria for analysis in this study included patients with singleton pregnancies at our institution who received greater than 50 percent of care with the generalist team. Exclusion criteria included those enrolled in Centering Pregnancy®, previable deliveries (less than 23 weeks), transfer into PodCare or initiation to prenatal care after 20-week gestation, and transfer to maternal-fetal medicine during prenatal care. Maternal-fetal medicine consultations were not included as an additional provider or visit.

We extracted data on the primary and secondary outcomes. Primary outcomes included number of prenatal care visits and number of providers seen. Secondary outcomes included gestational age at initiation of care and delivery, mode of delivery, infant weight, APGARS, and date of postpartum visit. Manual chart review was performed to verify completion of Group B streptococcus testing, glucose tolerance testing, and attendance at a postpartum visit.

Preterm delivery and low birth weight were defined as prior to 37 weeks and less than 2500 grams, respectively. Two HEDIS measures analyzed include percentage of deliveries receiving a postpartum visit and timeliness of care. A postpartum visit was defined as occurring on or between 21 and 56 days after delivery. Timeliness of care is defined as receiving care in the first trimester or within 42 days of enrollment in the organization [7]. The number of visits excludes the postpartum visit. Indications for early gestational diabetes screening included body mass index greater than 30 kg/m^2^ and a history of gestational diabetes.

Reliability was ensured by manual chart review. Excel was used to identify implausible data points, such as gestational age at delivery in excess of 42 weeks, and missing data, which were then corrected or obtained by chart review. The manual chart review was performed by the primary author (ED).

The data was tabulated comparing before and after the PodCare intervention. A *P* value of <0.05 (two-tailed) was regarded as significant. Data were analyzed in Excel and statistical comparisons made using OpenEpi and SPC XL. Categorical and continuous variables were analyzed using chi square and *t* test, respectively.

## 3. Results

A structured prenatal care schedule was implemented as detailed in Box 1 for all patients who entered PodCare after January 1, 2014. For the study period, of 450 patients, 188 patients (42%) chose the physician-led group in 2014, compared to 100 of 390 (26%) in 2013 (P<0.000001).

**Figure 1:**
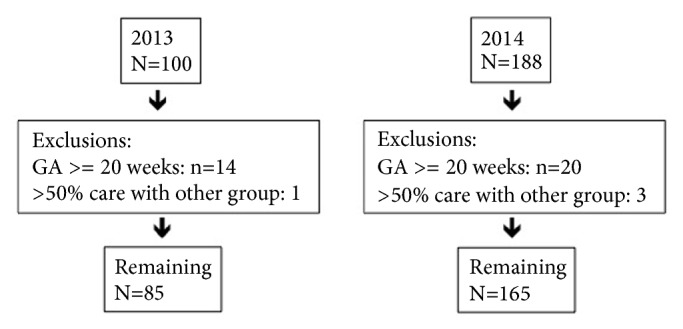
Exclusion criteria for study subjects.

After application of exclusion criteria, 85 women in 2013 and 165 women in 2014 were included in the study. Women were excluded who entered care after 20 weeks (14 in 2013 and 20 in 2014) and received greater than 50 percent of care with a different provider group (1 in 2013 and 3 in 2014), as detailed in Figure 1. Demographic information, including age, percent nulliparous, body mass index, insurance status, and race, was collected (Table 1).

**Figure 2:**
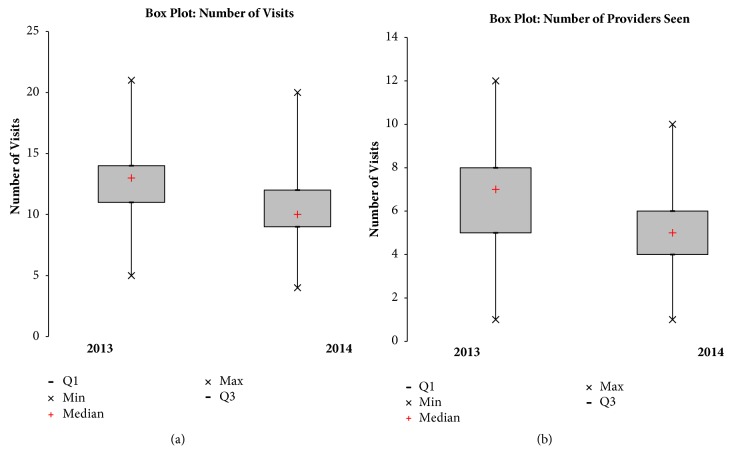
(a) The box plot demonstrates a decrease in the median number of providers seen. It also demonstrates a decrease in dispersion of the middle 50 percent. (b) The box plot demonstrates a decrease in the median number of visits.

The median number of visits per pregnancy decreased from 13 to 10 (p < 0.00004). The first and third quartile shifted from 11 to 9 and 14 to 12, respectively, with the interquartile range unchanged at 3 (Figure 2(a)). The median number of providers seen decreased from 7 to 5 (p < 0.0000008). The first and third quartile shifted from 5 to 4 and 8 to 6, respectively, with the interquartile range changing from 3 to 2 (Figure 2(b)).

Outcomes that were not statistically significant (Table 2) include preterm birth rate (8.2% in 2013, 10.9% in 2014), percentage of low birth weight infants (7.1% in 2013, 9.1% in 2014), indicated early diabetes screening (97.7% in 2013 and 97.6% in 2014), second trimester diabetes screening (96.5% in 2013 and 98.8% in 2014), and Group B streptococcus known at term delivery (100% in 2013 and 2014).

There were no statistically significant changes in mode of delivery (Table 2). Spontaneous vaginal delivery was 59% in 2013 and 64% in 2014 (p=0.40). Cesarean delivery rate was 38% in 2013 and 30% in 2014 (p=0.20). Operative vaginal delivery was 4% in 2013 and 6% in 2014 (p=0.45).

The percentage of woman attending a postpartum visit was unchanged at 93 percent for 2013 and 2014. The median gestational age for initiating care was during the 8th week of pregnancy both before and after the intervention. Under the PodCare model, 91 percent of women initiated care in the first trimester. Of the women who entered care after the first trimester (1 in 2013 and 13 in 2014), the majority entered PodCare as a transfer (1 in 2013 and 10 in 2014). Three subjects had their initial entry to care after the first trimester in 2014 compared to zero in 2013. We were unable to determine if women transferring care had a lapse of greater than 42 days in their care given limitations of the available data. There were no missing data points for the final analysis.

## 4. Discussion

PodCare is a value-based initiative that delivers high quality prenatal care with fewer visits and thus has the potential for lower cost. Woman participating in PodCare received care from fewer providers, which increased continuity of care. Timeliness of care and postpartum visit attendance remained excellent, especially when compared nationally. There were no statistically significant changes in the remaining maternal or neonatal outcomes. Importantly, the study may not have been powered to detect these differences.

PodCare successfully decreased the median number of prenatal visits per pregnancy to the target based on the structured schedule. This intervention also decreased the median number of providers seen. This is an important outcome given the goal of PodCare to increase continuity of care, especially in an academic setting where there are many challenges to continuity.

There is support for fewer prenatal appointments from other institutions, such as Kaiser Permanente, whose prenatal care guidelines include nine visits [8]. NICE guidelines from the United Kingdom features ten appointments for nulliparous women and seven appointments for multiparous women [9]. This more limited schedule has not been demonstrated to have any adverse outcomes.

Perinatal outcome measures, including HEDIS and Healthy People 2020 measures, remained excellent under the PodCare model. According to HEDIS, the national average for woman receiving a postpartum visit was 73.2% and 60.9% for commercial HMO and Medicaid in 2015, respectively. The timeliness of prenatal care was 83.7% and 80.0% for commercial HMO and Medicaid in 2015, respectively [10]. PodCare performs well above these national averages on both measures.

We hypothesize that the PodCare model allowed the generalist division to care for higher risk women throughout their pregnancy. This is due to the coordination of care at weekly Pod meetings that enabled associate providers to handle routine care for these women at appointments while ensuring that the attending physician approved the overall plan of care. During the study period, the overall census of the MFM team decreased by 6 percent, which makes it unlikely that high-risk patients were transferred at higher numbers to MFM care.

The comparison before and after the intervention contributes to internal validity; no other changes were made to prenatal care at the time of this intervention at our institution. One positive unintended consequence of PodCare is that associate providers are more likely to supervise junior residents, while also seeking guidance from senior residents. This allows senior residents to gain experience as a consulting physician, which is a valuable skill that meets the Accreditation Counsel for Graduate Medical Education Core Competency of Interpersonal and Communication Skills.

PodCare appears to be a popular model for prenatal care, as patients increasingly selected the physician team. By decreasing the number of visits per pregnancy, PodCare accommodated a greater volume of patients without increasing the number of providers or staff.

Strengths of this study include a washout period resulting in all analyzed patients receiving care under the intended model. Data obtained from the electronic medical record was verified by manual review, including outliers and missing data points. Weaknesses include that the study was not powered to detect adverse neonatal or maternal outcomes.

Bias in this study includes patients that were not randomized to the type of care they received. Two assumptions of the study are the facts that patients prefer to see fewer providers and that continuity can be measured by number of providers seen and used as a proxy for patient satisfaction.

This intervention to standardize individual prenatal care is generalizable to academic departments but might also be beneficial for group practices. One limitation to generalizability is that this patient population might be unique given very favorable adherence to care as demonstrated by glucose tolerance testing and attendance at the postpartum visit.

In a time of healthcare austerity, PodCare presents savings in opportunity cost given more available clinic visits for other obstetrical or gynecologic patients. If the PodCare model were universally adopted at Dartmouth-Hitchcock, there would be a savings of 2160 prenatal care visits assuming a volume of 1200 prenatal patients per year. The model also allows residents and associate providers to provide the bulk of care under the supervision of an attending physician. This is a cost-effective and resource-wise decision. Additional research could be performed to assess changes in provider and patient satisfaction after implementation of the PodCare model. Future research could also include analysis of cost-per-pregnancy under each model and defining more comprehensive quality indicators for prenatal care.

## Figures and Tables

**Box 1:**
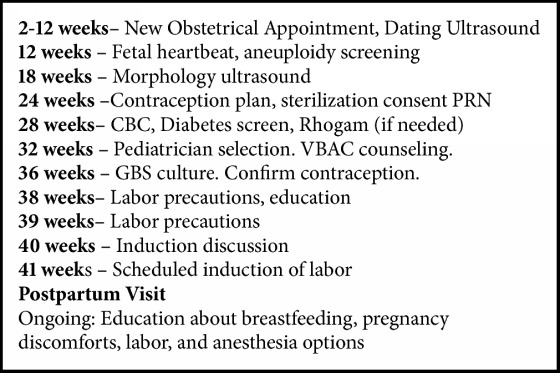
The structured prenatal care schedule implemented with PodCare.

**Table 1:** Demographics.

Characteristics	2013 Pre-PodCare (n=85)	2014 Post-PodCare (n=165)
Age (y)	30.9 (19.1-42.6)	30.1 (18.8-42.6)

Nulliparous	38 (45)	81 (49)

Ethnicity		
White	79 (93)	145 (87)
Black	1 (1)	1 (1)
Asian	5 (6)	16 (10)
Native American	-	1 (1)
Declines to List	-	2 (1)

Insurance Status		
Private	52 (61)	100 (61)
Public	12 (14)	28 (17)
Uninsured	21 (25)	36 (22)

BMI	26.1 (18.1-45.4)	26.4 (16.4-56.6)

BMI, body mass index (kg/m^2^).

Data are n (%) or mean (range).

**Table 2:** Secondary outcomes.

Secondary Outcome	2013: Traditional (n=85) *∗*	2014: PodCare (n=165) *∗*	P value
Mode of Delivery †			
Spontaneous vaginal	50 (59%)	106 (64%)	0.40
Cesarean	32 (38%)	49 (30%)	0.20
Operative vaginal	3 (4%)	10 (6)	0.45

Group B Strep known at term delivery	78 of 78 (100%)	147 of 147 (100%)	Not applicable

Indicated early diabetes screening ‡	14 of 16 (97.7%)	25 of 29 (97.6%)	0.73

28 week diabetes screening	82 of 85 (96.5%)	162 of 164 (98.8%)	0.85

Low birth weight infant **§**	6 of 85 (7.1%)	15 of 165 (9.1%)	0.58

Preterm delivery	7 of 85 (8.2%)	18 of 165(10.9%)	0.50

*∗* Dominators change by category based on included subjects.

† Percentages do not sum to 100 due to rounding.

‡ BMI >= 30kg/m^2^ or history of gestational diabetes.

§ Less than 2500 grams.

## Data Availability

The datasets generated during and analyzed during the current study are available from the corresponding author on reasonable request.

## Additional Points

PodCare increases the value of individual prenatal care by decreasing the number of visits, increasing continuity, and providing care coordination.
